# Marta L. Wayne

**DOI:** 10.1093/gbe/evac134

**Published:** 2022-09-13

**Authors:** Adri K Grow

**Affiliations:** Department of Biological Sciences, Smith College, Northampton, MA 01063, USA

We continue our biography section, featuring former Society for Molecular Biology & Evolution (SMBE) president Marta L. Wayne. The following is based on a March 2022 interview with Marta.

## How Did You Become a Scientist?

“This is always what I wanted to do,” says Marta L. Wayne, who is transitioning from Professor of Biology to serving as the Dean and Associate Provost for the University of Florida International Center. Growing up, Marta liked to watch the TV show *Mutual of Omaha’s Wild Kingdom*, a wildlife documentary hosted by both Marlin Perkins, who taught all about different animals, and Jim Fowler, who handled the animals. Marta wanted to be like Marlin one day. Marta was also influenced by her grandmother’s and mother’s passion for natural history and birdwatching and her father’s interests in gardening. A career in biology just made sense.

While in high school, Marta’s interests lay in natural history and evolutionary biology. Marta started taking classes at the LA County Museum of Natural History on the weekends and participated in exciting things like digging fossils, collecting fish, and exploring rivers. She also enjoyed talking with people who worked at the museum. Marta expressed her interests in evolution often and was told that evolution was a dying field (i.e., that someone would literally have to die for a job to become available) and that if she really liked evolution, she must also really like the booming field of genetics. From that point on, Marta went all-in; she focused her studies on genetics for her bachelor’s degree in biology at the University of California, San Diego and then she started a PhD in molecular biology at Princeton University. It was in graduate school when Marta learned that there was a whole-graduate department dedicated to ecology and evolutionary biology right alongside the department of molecular biology.

In Marta’s graduate program, she had to rotate through different labs to find one with overlapping interests and the right lab culture for her. Marta’s first rotation was in a lab that studied oncogenes and she found the lab fun and she knew this would be a great option. Because Marta was confident in her interests with that first lab, she was not worried about the selection of the second lab to rotate through and “frivolously” picked Marty Kreitman’s lab. Marta had met Marty during her interview for graduate school; he worked with fruit flies and evolution. Marta loved *Drosophila*, as she had worked with Dan Lindsley during her undergraduate studies. Everything about Marta’s second rotation piqued her interests and led her to stay in the Kreitman lab and ultimately to switch from getting a PhD in molecular biology to one in ecology and evolution.

## What Are Some Challenges You Have Faced in Your Career?

Marta openly shares that she failed her qualifying examinations as a PhD student and was close to departing from the graduate program. Although a deflating experience, this experience solidified the switch from the department of molecular biology to the department of ecology and evolution. In the new department, in which Marta admittedly had little background knowledge in having focused coursework on molecular biology, Marta had to again prepare for and take her qualifying examinations. Fortunately, going back to her initial love of evolution, Marta thoroughly enjoyed the assigned topics and reading material and passed her qualifying examinations. Marta shares this difficult time in her career as a way for students to understand that failures can happen, but that they can be overcome.

## What Do You Do for Fun?

Marta has many interests and hobbies that lie outside the sphere of science. Anyone who has ever worked with Marta would know that she enjoys cooking and entertaining. Marta also has a recent interest in quilting and sewing, but her absolute favorite thing to do is backpacking. While Marta was a student (both undergraduate and graduate), she loved taking non-science classes for much-needed intellectual inspiration. Marta has found that creative outlets and a broad knowledge base help her to think in novel ways about science. At the time of our interview, Marta was working toward a graduate certificate in Latin American Studies and over the past year has taken courses in sustainable development. This experience has been transformative as Marta integrates science and academic leadership, establishing trust in professional spaces.

## What’s Some Advice for People Entering the Field of Science?

Because rejection is common, whether it be a paper, a grant, or a position, Marta believes the key attributes of a scientist are stubbornness and resilience. It is important to be able to pick yourself up, know that your ideas are worth the work, and keep making progress. She also believes it is even more important to find your allies for guidance along the way. Marta also says having strong intuition and trusting your gut is important because oftentimes she has followed that intuitive feeling on risky projects that were very successful. In addition to resilience, Marta emphasizes that scientists need to have a good work-life balance because working in the lab for 14 h a day does not make you a better scientist, it just means that you spend more time in the lab. Being able to work efficiently and take breaks to recharge is essential for long-term success.

## Who Has Been Your Biggest Mentor or Influence on Your Career?

Marta has had three people in particular who have been the most influential during her career. The first was her undergraduate mentor Dan Lindsley, known as the father of *Drosophila* genetics, who set high standards and challenged Marta in incredibly supportive ways. The second is Marta’s doctoral advisor Marty Kreitman who has had the biggest impact on her life. Marty to this day remains a fantastic advocate, sounding board, and coach for Marta. The third is Marta’s postdoc advisor Trudy Mackay who showed Marta a different style of doing science when compared with what she had known previously. Trudy was much more hands-on, often finding Marta at the bench and asking about experiments and vials of fruit flies, which worked well for Marta’s process; she also modeled work-life balance with her passion for horses. Of course, there are many other people that Marta has crossed paths with that have been important at different times and she has been able to find a supportive community.

## Your Favorite Contribution to the Literature?

The first is “Statistical tests of neutrality in the age of weak selection” published in *Trends in Ecology & Evolution* in 1998; a review paper that Marta wrote with Katy L. Simonsen. This provides a nice background synthesis on the neutral theory and an overview of statistical tests used at the time written in an approachable and accessible manner. This is at the top of the list for Marta because this was a paper that flowed so organically and in some ways wrote itself, which made the whole process enjoyable. The second is “Combining mapping and arraying: An approach to candidate gene identification” published in *PNAS* in 2002 coauthored with Lauren M. McIntyre. Marta loves this paper because every once in a while she will see who has cited the paper in their studies and is then able to see how far the field has progressed. Within the first few years of publishing this paper, Marta saw firsthand how basic science gives rise to applied science. For example, the techniques described in the paper were adapted to identify malting quality in barley for beer making and to find genes connected to human hearing. Marta found that bench-to-industry connection very fun and rewarding.

## Any Final Thoughts?

Marta’s favorite thing about teaching is the students, particularly the fun in seeing where they go and what they end up doing. One particular story Marta shares is that of an undergraduate in Marta’s genetics class who ended up running a program to control the flu virus in Alachua county (where Gainesville, FL is located) through vaccination in schools. The goal was to reduce rates of hospitalization for all age groups by vaccinating school children; Marta remembers both suggesting edits for and completing consent forms for her own child to get vaccinated that would later be used in the study being conducted by one of her previous undergraduate students (Marta wants to be sure that you know that the Alachua County’s ControlFlu project was a resounding success!).

**Figure:**
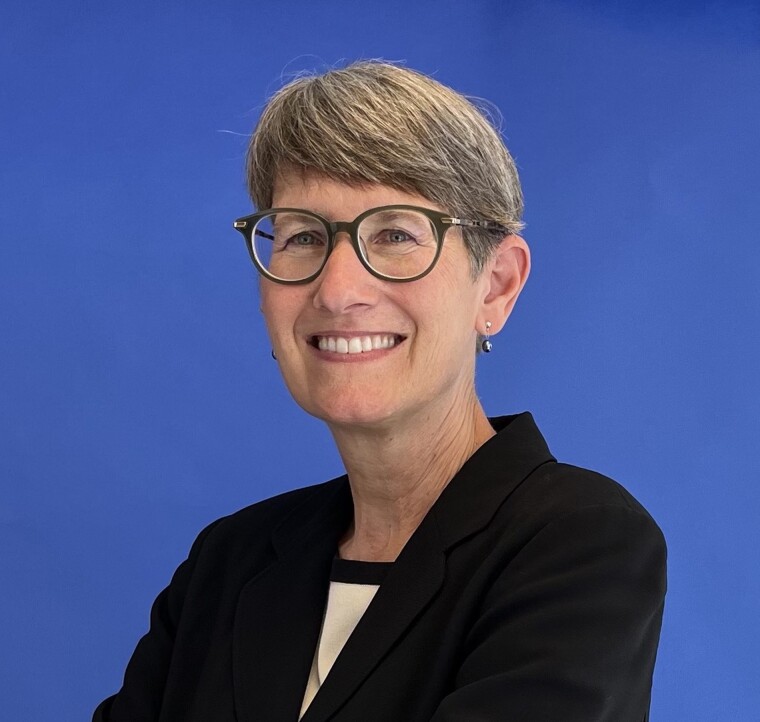
Dr Marta L. Wayne, Dean of the International Center and Associate Provost, University of Florida.

**Figure:**
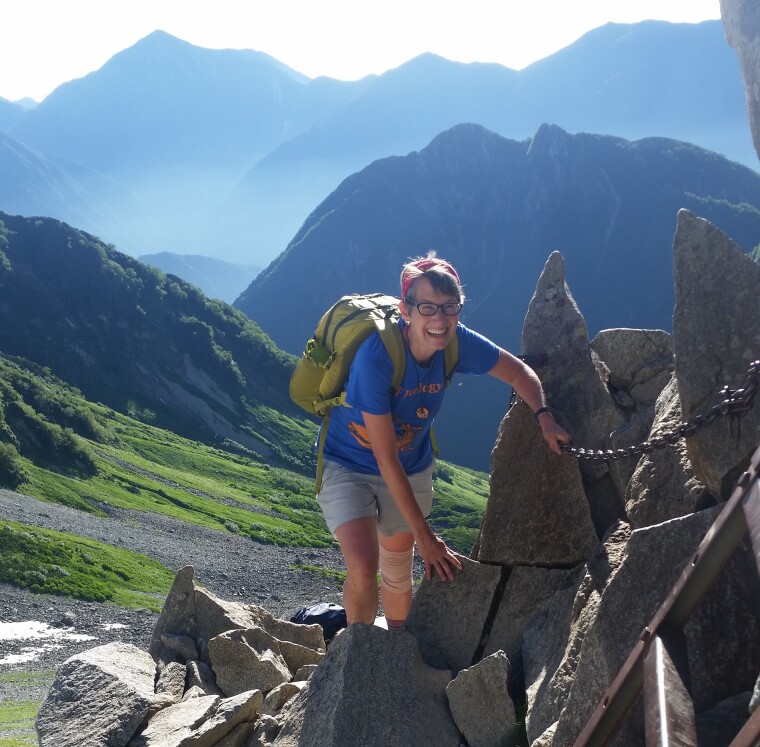
Marta, with Lulu Stader and Cathy Kennedy, on their way to Hotaka-dake-sanso in the Japanese Alps after the 2018 SMBE meeting in Yokohama.

